# Classification of Benign and Malignant Thyroid Nodules Using a Combined Clinical Information and Gene Expression Signatures

**DOI:** 10.1371/journal.pone.0164570

**Published:** 2016-10-24

**Authors:** Bing Zheng, Jun Liu, Jianlei Gu, Jing Du, Lin Wang, Shengli Gu, Juan Cheng, Jun Yang, Hui Lu

**Affiliations:** 1 Shanghai Institute of Medical Genetics, Shanghai Children’s Hospital, Shanghai Jiao Tong University, Shanghai, China; 2 Department of Laboratory Medicine, Renji Hospital, School of Medicine, Shanghai Jiao Tong University, Shanghai, China; 3 Department of Otolaryngology, Renji Hospital, School of Medicine, Shanghai Jiao Tong University, Shanghai, China; 4 Department of Otolaryngology-Head and Neck Surgery, Xinhua Hospital, School of Medicine, Shanghai Jiaotong University, Shanghai, China; 5 Ear Institute, Shanghai Jiaotong University, Shanghai, China; 6 Key Laboratory of Molecular Embryology, Ministry of Health and Shanghai Key Laboratory of Embryo and Reproduction Engineering, Shanghai, China; 7 Department of Ultrasonography, Renji Hospital, School of Medicine, Shanghai Jiao Tong University, Shanghai, China; 8 Department of Bioengineering, University of Illinois at Chicago, Chicago, Illinois, United States of America; 9 Department of Ultrasonography, Xinhua Hospital, School of Medicine, Shanghai Jiao Tong University, Shanghai, China; National Research Council, ITALY

## Abstract

**Background:**

A key challenge in thyroid carcinoma is preoperatively diagnosing malignant thyroid nodules. A novel diagnostic test that measures the expression of a 3-gene signature (*DPP4*, *SCG5* and *CA12*) has demonstrated promise in thyroid carcinoma assessment. However, more reliable prediction methods combining clinical features with genomic signatures with high accuracy, good stability and low cost are needed.

**Methodology/Principal Findings:**

25 clinical information were recorded in 771 patients. Feature selection and validation were conducted using random forest. Thyroid samples and clinical data were obtained from 142 patients at two different hospitals, and expression of the 3-gene signature was measured using quantitative PCR. The predictive abilities of three models (based on the selected clinical variables, the gene expression profile, and integrated gene expression and clinical information) were compared. Seven clinical characteristics were selected based on a training set (539 patients) and tested in three test sets, yielding predictive accuracies of 82.3% (n = 232), 81.4% (n = 70), and 81.9% (n = 72). The predictive sensitivity, specificity, and accuracy were 72.3%, 80.5% and 76.8% for the model based on the gene expression signature, 66.2%, 81.8% and 74.6% for the model based on the clinical data, and 83.1%, 84.4% and 83.8% for the combined model in a 10-fold cross-validation (n = 142).

**Conclusions:**

These findings reveal that the integrated model, which combines clinical data with the 3-gene signature, is superior to models based on gene expression or clinical data alone. The integrated model appears to be a reliable tool for the preoperative diagnosis of thyroid tumors.

## Introduction

The incidence of thyroid carcinoma has substantially increased in the United States in recent years. In 2008, the estimated number of new cases of thyroid cancer was 37,340, in contrast to the estimated 60,220 new cases in 2013, which indicates that the incidence nearly doubled in five years [1, 2]. This dramatic increase in the number of new cases is mainly the result of the gradual increase in the use of ultrasound in routine physical examinations [3, 4].

A key challenge in thyroid cancer research lies in distinguishing benign thyroid nodules from malignant tumors [5–7]. The problem is that most thyroid nodules are benign, with only 5–15% being malignant. The current cornerstone of preoperative thyroid nodule character evaluation is ultrasonographically guided fine needle aspiration (FNA). However, a clear limitation of this approach is that approximately 15–30% of FNAs reveal indeterminate or suspicious cytological findings [8]. Moreover, only one-fifth of the indeterminate FNAs are found to be malignant after diagnostic surgical operations [7]. Therefore, there is a compelling case for developing more practical and accurate diagnostic methods to preoperatively evaluate the characteristics of thyroid nodules, which could play an important role in the management of patients with benign lesions to avoid unnecessary thyroid lobectomies.

We hypothesis that the integration of small gene signatures and clinical data would substantially impact the predictive accuracy of thyroid tumor models and have promise or application in routine clinical practice. First, clinical data, including patient history, medical examination results and ultrasound imaging analysis, are typically available and form the foundation of day-to-day clinical decisions. Furthermore, data on clinically important variables have the distinct advantage of a relatively low noise level, which is important for cancer prediction accuracy.

Second, gene expression profiles have demonstrated the power to help determine the heterogeneity of various tumors [6, 9, 10]. Even patients with similar symptoms may have a distinct treatment response or prognosis, which is the foundation for advocating personalized medicine. Therefore, molecular biomarkers may offer an alternative means to indicate tumor behavior and enhance the predictive ability of models integrating multiple forms of clinical data. However, it is essential to select a small number of genes that can be easily assayed via quantitative PCR (qPCR) to predict thyroid tumors in clinical applications rather than to use a microarray analysis, which is more complex, expensive, and hard to interpret [11].

In our previous study, we identified a three-gene signature—*DPP4*, *SCG5* and *CA12*—that is measured via qPCR and performed well in distinguishing benign and malignant thyroid nodules[12]. Consistent with the integrative viewpoint, our present analysis focuses on the selection of relevant clinical information to develop an integrated clinico-genomic model for further improvement of thyroid cancer prediction.

## Materials and Methods

### Patient clinical information and tissue samples

To select and validate clinical variables that are significantly correlated with thyroid tumor malignancy, we collected data from 771 patients with clinically significant thyroid nodules who had undergone thyroid ultrasonography and were ultimately diagnosed with a thyroid tumor after partial or total thyroidectomy operations. The patients had received treatment in 2011–2012 at the Otolaryngology Department in Shanghai Renji Hospital, China. The mean age ± standard deviation of the 771 patients, including 202 males (26.2%) and 569 females (73.8%), was 47.4 ± 12.5 years (range: 13–77 years). Thyroid ultrasonography examinations were performed and recorded by two radiologists who were professionals in thyroid sonography using a L12-5 transducer (Siemens, Germany) on an S2000 scanner (Siemens, Germany). Two additional independent cohorts, which include 70 (Cohort 2) [12] and 72 (Cohort 3) randomly selected patients in 2013 from two centers (Shanghai Renji Hospital and Xinhua Hospital), were analyzed for validation of the model based on clinical information and an integrated model that combined clinical information with gene expression. Tissue samples preserved in the RNALater reagent during the operation and immediately transferred to -80°C to stabilize the RNA, together with the clinical data collected from the 142 patients. The demographic, clinical and ultrasound characteristics for each patient in the three cohorts are summarized in Table 1. The distributions of the histopathological subtypes of benign and malignant thyroid nodules in the three cohorts are shown in Table A in S1 File. All patient personal information was de-identified and is unknown to the authors, and the sample collections were approved by the institutional review boards of the Renji and Xinhua Hospitals.

**Table 1:** Demographic, clinical and ultrasound characteristics of 913 patients.

Characteristics	Recorded variables
Demographics	Age at last birthday
	Sex: male/female
Clinical	Course of disease: from nodule detection to operation
	Nodule increase
	Mobility of mass in physical examination: mobile/fixed/nonpalpable
	Texture of mass upon physical examination: soft/medium/hard
Ultrasound	Nodule maximum size: <1.00 cm/1.00–1.99 cm/2.00–2.99 cm/≥3 cm Taller-than-wide sign (anteroposterior dimension / transverse dimension): <1/≥1 Maximal nodule area
	Cystic lesion
	Nodule number: single/germination/multiple Nodule position: one side/two sides
	Nodule boundary: clear/vague
	Nodule echoes: homogeneous/inhomogeneous
	Nodule echo type: hypoechoic/isoechoic/hyperechoic/mixed echogenicity
	Nodule morphology: regular/irregular
	Nodule peripheral vessels: abundant/scarce
	Lymphadenopathy number
	Lymph node morphology: regular/irregular
	Lymph node boundary: clear/vague
	Lymph node peripheral vessels: abundant/scarce
	Lymph node structure: damaged/undamaged
	Calcification: null/calcification /micro calcification

### RNA extraction and qPCR

Extraction and analysis of RNA were performed as previously described (our paper). To validate the expression levels of the three genes—*DPP4*, *SCG5* and *CA12* (which were selected by a Bayesian model averaging (BMA) algorithm in our previous research and predict thyroid tumors with good accuracy), reverse transcription real-time qPCR was performed using the SYBR Green method and the previously reported primer sets [12]. Glyceraldehyde-3-phosphate dehydrogenase (GAPDH) was used as a reference gene, The relative gene expression level was calculated as follows: 2^-ΔCt^×100 (ΔCt = Ct _target gene_−Ct _GAPDH_) [13].

### Feature selection in clinical data using random Forest (RF) models

Five hundred and thirty-nine patients (70% of the patients in Cohort 1; data set: Cohort 1-TR), which included 241 benign and 298 malignant samples, were randomly stratified as the training set to construct the prediction model. Two hundred and thirty-two patients (30% of the patients in Cohort 1; data set: Cohort 1-TE), which included 104 benign and 128 malignant nodules, comprised the test data.

The original RF model contains 23 variables (Table 1), including eleven categorical variables: (1) Sex (male = 0, female = 1), (2) Mobility of mass upon physical examination (nonpalpable = 0, fixed = 1, mobile = 2), (3) Texture of mass upon physical examination (nonpalpable = 0, soft = 1, medium = 2, hard = 3), (4) Max nodule diameter in ultrasound (<1.00 cm = 1, 1.00–1.99 cm = 2, 2.00–2.99 cm = 3, **≥** 3 cm = 4), (5) Nodule number in ultrasound (single = 1, germination = 2, multiple = 3), (6) Nodule echo type in ultrasound with respect to normal thyroid parenchyma (hypoechoic = 1, isoechoic = 2, hyperechoic = 3, mixed echogenicity = 4), (7) Ultrasound lymph node number (without lymph node involvement = 0, single lymph node = 1, two lymph nodes = 2, multiple lymph nodes = 3), (8) Ultrasound lymph node morphology (without lymph node involvement = 0, morphology regular = 1, morphology irregular = 2), (9) Ultrasound lymph node boundary (without lymph node involvement = 0, boundary clear = 1, boundary vague = 2), (10) Ultrasound lymph node veins (without lymph node involvement = 0, veins abundant = 1, veins scarce = 2), and (11) Calcification (No calcification = 0, calcification = 1, micro-calcification = 2). The ultrasound images in our study were reviewed by two experienced sonographers (J.D. and L.W) with more than 5 years’ experience. As for the inconsistent results, the two examiners with another expert examiner (J.X.) who has more than 15 years’ experience would review and reach a consensus by discussion.

The random forest algorithm [14] has commonly been used for descriptor selection because it provides information regarding variable importance for the classifier. The percent increased mean square error (%IncMSE) was calculated, which indicates the declension of the predictive ability of the model when each descriptor is permuted in turn by random noise. An increased %IncMSE typically indicates a greater role for the variable in the prediction model. In this study, we set the cut-off value of %IncMSE to 10, which indicates that if the %IncMSE value of a descriptor is 10 or higher, it can be selected as a variable to distinguish benign from malignant nodules.

### Data mining algorithm based on clinical data and cross-validation

The most commonly used classification methods can be divided into two groups: one category comprises the strong classification algorithms, such as support vector machine (SVM) and Naïve Bayes (NB); the other category comprises the combination classification algorithms, which are also referred to as weak classification algorithms, such as random forest (RF). SVM and RF are the most widely used classifiers in bioscience; thus, comparisons between the performances of these two methods have been performed many times [15–18]. RF is a useful classifier with the following unique advantages over SVM: it provides the importance of the variable, exhibits good tolerance to noise in the data and little or no overfitting and is applicable to several data types. Thus, we employed RF for classification analyses in this study. The RF algorithm was implemented by the R package ‘randomForest’[19] with its default parameters was applied.

**Fig 1:**
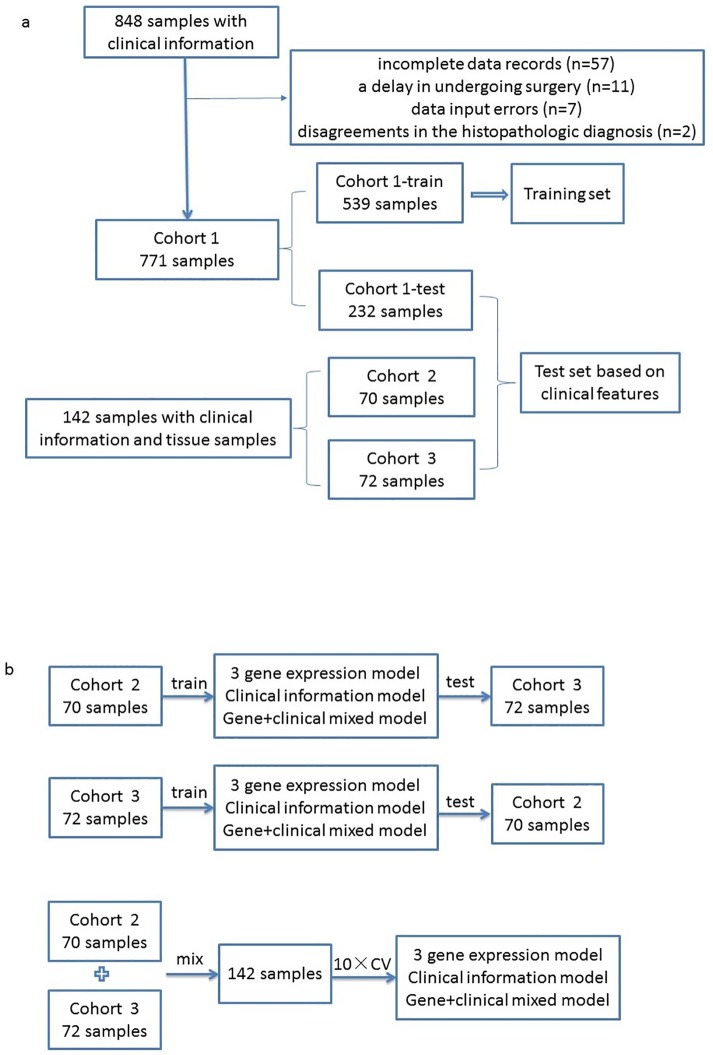
Workflow of this study. (a) Flow diagram of feature selection and validation of clinical data. Cohort 1 comprised 771 samples that were randomly divided into the training (539 samples) and test (232 samples) sets. Two additional independent data sets, Cohorts 2 and 3, included 70 and 72 samples, respectively, from Renji and Xinhua Hospital and were also employed as test sets to validate the predictive accuracy of the classification based on clinical data. (b) Flow diagram for the comparison between the classifier models based on the three gene expression levels, the clinical information, and integrating the gene expression with clinical data.

The arrangement of the training and test sets to construct and validate the model based on clinical information is summarized in Fig 1. Ten-fold cross-validation in the training set was used to assess the robustness and the predictive results of the models. Thus, the training set was randomly split into 10 equal parts, in which 9/10 of the data were used to develop the model and the remaining 1/10 of the data were evaluated as the independent test data. Furthermore, three independent data sets, including Cohort 2 [12], Cohort 3 and 30% of the samples randomly selected from Cohort 1, comprised the test set. The sensitivity, specificity, positive predictive value (PPV), negative predictive value (NPV) and predictive accuracy were calculated to evaluate the predictive performance.

### Integrated clinical-gene-expression models and cross-validation

One hundred and forty-two thyroid tissue samples, including 70 samples from Cohort 2 and 72 samples from Cohort 3, had both clinical and gene expression information. The relative mRNA expression levels of the three genes (*DPP4*, *SCG5* and *CA12*) measured by real-time qPCR, the clinical predictive factors previously described, and the combined gene signature and clinical variables were applied to the random forest classification with default parameters. The information obtained from Cohorts 2 and 3 was from two centers; thus, one cohort was used as the training set, and the other cohort was used as the test set. In addition, 10-fold cross-validation of the 142 samples (together with Cohorts 2 and 3) was conducted to assess the integrated model. The work flow for the comparison of the classifier models based on the three-gene signature, the clinical information, and the integration of gene expression with clinical data is summarized in Fig 1b.

## Results

### Univariate analysis

The study population of Cohort 1, which included 848 patients, was recruited between January 2011 and December 2012 at Shanghai Renji Hospital to assess the important clinical characteristics in thyroid cancer prediction. Certain patients were excluded because of incomplete data records (n = 57), a delay in undergoing surgery (n = 11; more than 120 days from ultrasound examination), data input errors (n = 7), and disagreements in the histopathologic diagnosis (n = 2). Thus, 771 patients (90.9%) were included and were randomly divided into the training set (Cohort 1-TR, 539 samples, 241 benign nodules and 298 malignant tumors) and the test set (Cohort 1-TE, 232 samples, 104 benign nodules and 128 malignant tumors). Furthermore, the clinical data from two independent data sets, which included Cohort 2 [12] and Cohort 3, were recorded as two additional test sets for model validation (Fig 1). Data from 539 samples from data set Cohort 1-TR (70%) were available for feature selection and for the development of the clinical data cancer prediction model. All the relevant data for Cohort 1, 2 and 3 was available in S2 File.

To evaluate the potential risk factors involved in diagnosing malignant thyroid tumor, univariate analyses of the demographic data, physical examination and ultrasound-based variables in the training set were performed; the abbreviations for the clinical variables are listed in Table 2 (p<0.01 for all parameters). All variables except the two ultrasound-based variables (nodule number and position) were significantly different between benign and malignant thyroid nodules.

**Table 2:** Comparison of the characteristics of benign and malignant tumors in the training set following histologic classification of thyroid nodules.

Variable	Abbreviation	Training set (N = 539)		*p* value
		Benign (n = 241)n (%)	Malignant (n = 298)n (%)	
**Demographics**				
Age (years, median)*	Age	52	45.5	< .001
Sex	Sex			0.008
Male		59 (24.5)	89 (29.9)	
Female		182 (75.5)	209 (70.1)	
**Clinical**				
Course of disease (months, median)*	Medical_his	6	3	< .001
Nodule increase present	nodule_increase	111 (46.1)	92 (30.9)	< .001
Mobility of mass in physical examination	ME_L_mob			< .001
Nonpalpable		25 (10.4)	28 (9.4)	
Fixed		3 (1.2)	35 (11.7)	
Mobile		213 (88.4)	235 (78.9)	
Texture of mass in physical examination	ME_L_char			< .001
Nonpalpable		24 (10.0)	30 (10.1)	
Soft		137 (56.8)	99 (33.2)	
Medium		68 (28.2)	96 (32.2)	
Hard		12 (5.0)	73 (24.5)	
**Ultrasound**				
Max nodule diameter	US_N_max_D			< .001
<1.00 cm		26(10.8)	64(21.5)	
1.00–1.99 cm		89(36.9)	132(44.3)	
2.00–2.99 cm		68(28.2)	70(23.5)	
≥ 3 cm		58(24.1)	32(10.7)	
Taller-than-wide sign (≥1)	US_N_LW_ratio	45(18.7)	85(28.5)	0.008
Nodule maximum area (mm^2^, median)*	US_N_max_area	276	169.5	0.002
Cystic lesion present	US_cystic_lesion	60 (24.9)	6 (2.0)	< .001
Nodule number	US_N_num			NS
Single		78 (32.4)	102 (34.2)	
Germination		40 (16.6)	64 (21.5)	
Multiple		123 (51.0)	132 (44.3)	
Nodule position	US_N_pos			NS
One side		97(40.2)	128(43.0)	
Two sides		144(59.8)	170(57.0)	
Nodule boundary clear	US_N_bou	203 (84.2)	167 (56.0)	< .001
Nodule echoes homogenous	US_L_echoes	86 (35.7)	28 (9.4)	< .001
Nodule echo type	US_echoe_type			< .001
Hypoechoic		90 (37.4)	240 (80.5)	
Isoechoic		21 (8.7)	14 (4.7)	
Hyperechoic		3 (1.2)	3 (1.0)	
Mixed echogenicity		127 (52.7)	41 (13.8)	
Nodule morphology regular	US_L_mor	199 (82.6)	121(40.6)	< .001
Nodule peripheral vessels abundant	US_L_vas	62 (25.7)	159 (53.4)	< .001
Lymph node number	US_LN_num			< .001
None		211(87.6)	217(72.9)	
Single		2(0.8)	13(4.3)	
Germination		1(0.4)	4(1.3)	
Multiple		27(11.2)	64(21.5)	
Lymph node morphology regular if present	US_LN_mor	25/30	56/81	< .001
Lymph node boundary clear if present	US_LN_bou	27/30	64/81	< .001
Lymph node peripheral vessels abundant if present	US_LN_vas	7/30	13/81	< .001
Lymph node structure undamaged if present	US_LN_inner_str	28/30	61/81	< .001
Calcification	US_cal			< .001
No calcification		149 (61.9)	86 (28.9)	
Calcification		69 (28.6)	102 (34.2)	
Micro-calcification		(9.5)	110(36.9)	

NS, not statistically significant (P>0.05)

*These variables are listed as the median. Student’s t test was used for comparison between two groups. Other parameters were compared by *χ*^2^-test or Fisher’s test.

### Feature selection of clinical data by random Forest

The feature selection procedure was completed using the R package ‘randomForest.’ There are two main parameters in random Forest: **mtry**, which represents the number of different descriptors tried at each split; and **ntree**, which represents the number of trees in the forest. For the **ntree** parameter, we employed default values (**ntree** = 500); for **mtry**, we utilized a 10-fold cross-validation to test the cross-validated prediction performance of the models with a sequentially reduced number of predictors. As shown in S1 Fig, when **mtry** = 7, the model attained the optimum **mtry** with the lowest MSE at each step. Because the %IncMSE is consistent with the importance of the descriptors, we determined the most important parameters based on the %IncMSE values (S2 Fig). We employed the top seven features as the significant variables with a cutoff of ten for the %IncMSE value to train the clinical model, which included five ultrasound variables (Nodule morphology, Echo type, Calcification, Cystic lesion and Nodule boundary), one demographic variable (Age) and one physical examination variable (Texture of mass in physical examination).

### Validation of the predictive performance of the clinical information model

Seven variables were employed in the reduced random forest model with the training set of Cohort 1-TR, and three independent data sets were evaluated as the validation data set. For the test set Cohort 1-TE, which comprised 232 samples, the clinical information classifier accurately recognized 107 of the 128 malignant thyroid nodules, a sensitivity of 83.6% (95% CI, 77.2–90.0), and 84 of the 104 benign nodules, a specificity of 80.7% (95% CI, 73.2–88.3). The predictive accuracy was 82.3% (95% CI, 77.4–87.2). For the test set Cohort 2, which comprised 70 samples, the model correctly identified 23 of the 31 malignant samples, a sensitivity of 74.2% (95% CI, 58.8–89.6), and 34 of the 39 benign samples, a specificity of 87.1% (95% CI, 76.7–97.7). The predictive accuracy was 81.4% (95% CI, 72.3–90.5). For the test set Cohort 3, which comprised 72 samples from Xinhua Hospital, the classifier discriminated 29 of the 34 malignant nodules, a sensitivity of 85.3% (95% CI, 73.4–97.2), and 30 of the 38 benign samples, a specificity of 78.9% (95% CI, 66.0–91.9). The predictive accuracy was 81.9% (95% CI, 73.1–90.8). The predictive performances on the three data sets are summarized in Table 3 and indicate that the model with the 7 clinical characteristics was effective at distinguishing malignant thyroid nodules from benign nodules.

**Table 3:** Predictive performance of three independent data sets using the clinical information model.

Test set	Sensitivity (%)	Specificity (%)	PPV (%)	NPV (%)	Accuracy (%)
Cohort 1-TE	83.6	80.7	84.3	80.0	82.3
Cohort 2	74.2	87.1	82.1	81.0	81.4
Cohort 3	85.3	78.9	78.4	85.7	81.9

PPV: positive predictive value; NPV: negative predictive value.

### Combined model for thyroid tumor prediction

Although the predictive performance based on the 7 clinical variables was good in the random forest algorithm, a subset of patients with malignant tumors would still be misclassified into the benign group because of similar clinical symptoms. This finding suggests that gene expression level variables should be integrated to further improve the predictive accuracy and to analyze the mechanism of thyroid tumor at the gene level. In the combined model, the expression levels of the three genes selected by an iterative BMA method in our previous study [12] was combined with 7 clinical variables and simultaneously entered into the model classification.

**Fig 2:**
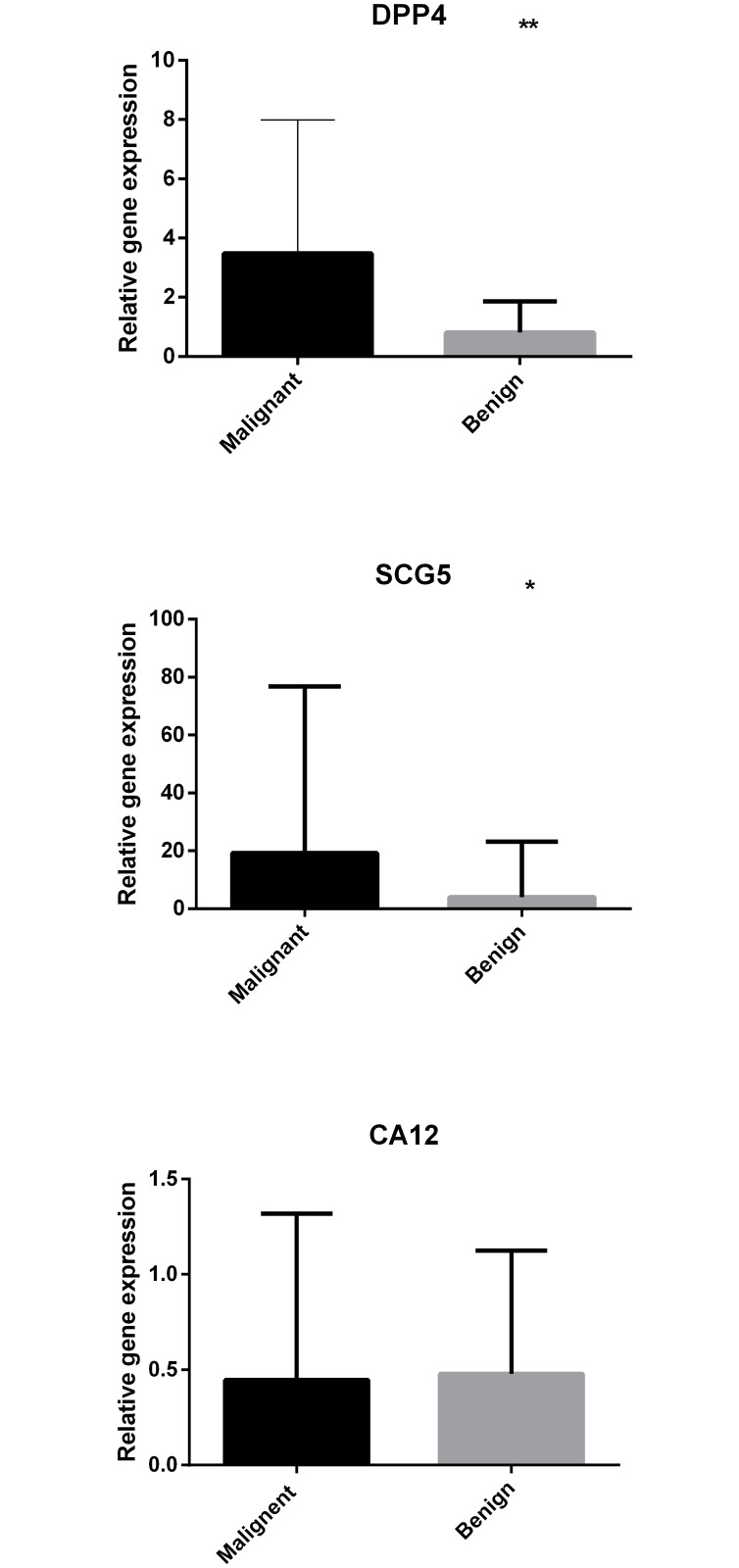
Histogram of the relative gene expression levels of *DPP4*, *SCG5* and *CA12* in malignant and benign thyroid nodules. **P<0.01 by two-tailed t test between benign and malignant thyroid tumor types. *P<0.05 by two-tailed t test between benign and malignant thyroid tumor types.

The gene expression levels of *DPP4*, *SCG5* and *CA12*, which were measured by qPCR in thyroid tissue samples, combined with the 7 selected significant clinical variables were detected and recorded for 142 patients, including 70 patients in Cohort 2 and 72 patients in Cohort 3. The relative gene expression levels of all the 142 patients are summarized in Table B in S1 File and Fig 2. In Cohorts 2 and 3, the gene expression levels of *DPP4* and *SCG5* were significantly different, whereas *CA12* exhibited little expression difference between benign and malignant thyroid nodules; these findings are consistent with our previous research [12].

The two data sets originated from different centers. Thus, Cohorts 2 and 3 were utilized as training and test sets, respectively, to compare the predictive performances of the random Forest model based on the three-gene signature, the 7 clinical variables, and the combination of gene expression and clinical data (Fig 1b). To further validate the superiority of the model combining clinical information and gene expression, we combined Cohorts 2 and 3 and performed a 10-fold cross-validation for the 142 patients. The input variables comprised the 3-gene signature, 7 clinical variables, and the combined gene expression and clinical data. The predictive performance was evaluated according to the sensitivity, specificity and predictive accuracy. As shown in Table 4, the predictive sensitivity using clinical data was lower than that of the model based on the expression levels of the three genes; however, the specificity of the prediction based on the clinical data was superior to that of the other two models. These findings indicate that the predictive abilities of gene expression and clinical data complement each other. Regardless of the cohort used as the test set or in the 10-fold cross-validation, the predictive accuracy was highest when the model was based on the combination of gene expression and clinical data.

**Table 4:** Comparison of thyroid cancer predictive performance based on the gene expression, clinical data, or integrated model.

Test set	Variables	Sensitivity(%)	Specificity(%)	Accuracy(%)
Cohort 3^a^	3 gene^d^	85.3	57.9	70.8
	7 Clinical inform^e^	79.4	73.7	76.4
	Gene+Clinical^f^	88.2	68.4	77.8
Cohort 2^b^	3 gene	87.1	77.0	81.4
	7 Clinical inform	74.2	92.3	84.3
	Gene+Clinical	93.54	84.6	88.6
Cohort2+ Cohort 3^c^	3 gene	72.3	80.5	76.8
	7 Clinical inform	66.2	81.8	74.6
	Gene+Clinical	83.1	84.4	83.8

^a.^Cohort 2 was used as the training set, and Cohort 3 was employed as the test set.

^b.^Cohort 3 was used as the training set, and Cohort 2 was employed as the test set.

^c.^Cohorts 2 and 3 were combined as a data set and validated by 10-fold cross-validation.

^d.^Model was developed based on the expression of *DPP4*, *SCG5*, and *CA12*.

^e.^Model was developed based on 7 significant clinical features.

^f.^Model was developed based on 10 input variables, including the expression levels of 3 genes and 7 significant clinical features.

## Discussion

The diagnosis of thyroid cancer remains a common problem in routine physical examination. It is important to preoperatively identify malignant thyroid nodules. With the development of genomic technology and advances in analyzing complex biomedical information, many investigators have focused on the molecular classification of thyroid nodules based on oligonucleotide microarray gene expression patterns using tissues or FNA samples [5–7, 20, 21]. However, the use of these methods for the prediction of thyroid nodule malignancy in routine clinical practice is impractical because 1) the quantitative measurement of vast, complex microarray gene expression patterns increases the cost for patients with thyroid nodules, and most genes are irrelevant to thyroid nodule characteristics [22]; 2) the analysis of a large number of genes requires clinical specialists to master complex statistical or computational tools [22–24]; 3) the results of microarrays may exhibit poor reproducibility and significant interbatch variability [11]; and 4) the various microarray platforms lead to differences in gene expression profiling [25].

To some extent, clinical data can address the deficiencies of genomic data. The routine clinical data used to aid in the diagnosis of thyroid tumors include the demographic data, physical examination data and ultrasound characteristics of patients with thyroid nodules. Recent studies have been conducted to distinguish malignant thyroid nodules according to characteristics evaluated by high-resolution thyroid ultrasonography [26–29]. Currently, microcalcifications, irregular nodule morphologies, hypoechogenicity of the nodules, and a blurred margin and shape have demonstrated strong relationships with malignant thyroid neoplasms [26–29]. Unfortunately, there are no uniform sonographic criteria for accurately predicting malignancy in thyroid nodules. Benign and malignant thyroid nodules can be difficult to clinically differentiate owing to their overlapping ultrasound patterns and the differences in the sensitivity of contrast-enhanced ultrasound in various ultrasonic testing devices. Thus, in addition to the ultrasonographic characteristics, other clinical data, such as the hormonal status or palpation of the nodules, should be integrated to further improve the accuracy of predicting malignancy in thyroid nodules.

In our study, 23 clinical variables that were considered to have correlations with malignant thyroid tumors were selected by the consensus of senior otolaryngologists and sonologists. A RF method was utilized to simplify the variables in the prediction model, and 7 variables were selected to distinguish between the benign and malignant groups in the training set, which included 1 demographic, 1 clinical and 5 ultrasound variables. Several features, including (1) irregular nodule morphologies, (2) nodules with hypoechogenicity, (3) nodules with calcification or micro-calcifications, (4) a lack of cyst formation, (5) patients at a young age, (6) palpate hard nodules upon physical examination, and (7) unclear boundaries of a nodule, were vital characteristics of malignant thyroid tumors and were included in our prediction model based on the clinical data. The distributions of the 23 descriptors were compared between the benign and malignant thyroid nodules. There were no significant differences in the number or position of the nodules between the benign and malignant thyroid tumors. Apart from these two characteristics, the remaining variables comprised risk factors for malignant tumors.

The model based on the 7 clinical variables performed well and was stable. In three independent data sets from two centers, the predictive accuracies were all greater than 80% using a random forest model, a value superior to those reported in previous studies that only used ultrasound parameters to predict thyroid nodule characteristics [26, 28–31]. Therefore, we are optimistic that ultrasound examination integrated with other demographic or clinical data will facilitate the development of a model superior to the application of ultrasound diagnosis alone.

Our study further confirms previous evidence that predictive accuracy can be enhanced by integrating clinical variables with genomic data [32–35]. However, the high cost, equipment, analytical procedures and critical need for precision in the operating steps to obtain reproducible results has restricted the application of microarray analysis in daily clinical use [36–38]. In contrast, qPCR is a practical and economic method in routine clinical use that can measure the fold changes in the expression levels of individual genes with high sensitivity and reproducibility [36, 39]. Therefore, in our previous study [12], we analyzed the public microarray data sets from the Gene Expression Omnibus, selected specific genes from overall gene expression profiling and constructed a qPCR-based model that included a three-gene signature (*DPP4*, *SCG5* and *CA12*).

To our knowledge, there are no publicly available databases that contain both gene expression data and relatively complete clinical information for thyroid tumors. In the current study, we measured the expression of the three previously identified genes via qPCR in 142 patients from two centers and then combined these data with 7 clinical factors selected in the current research to construct an integrated model to predict malignant thyroid nodules. To further reduce the impact of the unbalanced distribution from the two centers, we utilized a 10-fold cross-validation in addition to a 2-fold cross-validation (one data set as the training set and the other data set as the test set). It is clear that regardless of the validation set that was used, the best predictive performances were achieved by the random forest model based on the integrated clinical and molecular variables, which outperformed models that used clinical or genomic data alone. The gene expression model had a higher sensitivity than the clinical information model but a relatively low specificity in our study, and the clinical information model showed the reverse performance. Thus, in combination, these methods complement each other to produce an increased predictive accuracy.

However, we should note that this study comprised a pilot study. With advances in molecular medicine and improvements in personalized medical databases, more complex clinical factors and other clinical factors (such as the characteristics of ultrasound elastography or the levels of other serum biomarkers of thyroid tumors) and omics data (such as single-nucleotide polymorphisms, protein pattern concentrations, and metabolite analysis) may be further studied to improve the model and facilitate its day-to-day clinical application in thyroid cancer management.

## Supporting Information

S1 Fig Variations in the corresponding vector of error (error.cv) when different numbers of variables were employed at each split. (TIF)

S2 Fig Percent increased mean square error (%IncMSE) of the variables calculated by the random Forest analysis. (TIF)

S1 File Supporting tables. Table A. Histopathological subtype distribution in three cohorts. Cohort 1 comprised 711 patients from Renji Hospital with clinical information. Cohort 2 comprised 70 patients from Renji Hospital with clinical and gene expression information. Cohort 3 comprised 72 patients from Xinhua Hospital with clinical and gene expression information. Table B. Summary of the qPCR results for the expression of DPP4, SCG5 and CA12 in Cohort 2 and Cohort 3.
(DOCX)

S2 File Relevant data underlying the results described in manuscript. (XLSX)
