# Supplementary figures and images for: Classification of Benign and Malignant Thyroid Nodules Using a Combined Clinical Information and Gene Expression Signatures
Source: PLoS One. 2016 Oct 24;11(10):e0164570. doi: 10.1371/journal.pone.0164570 (PMC5077123; doi:10.1371/journal.pone.0164570)

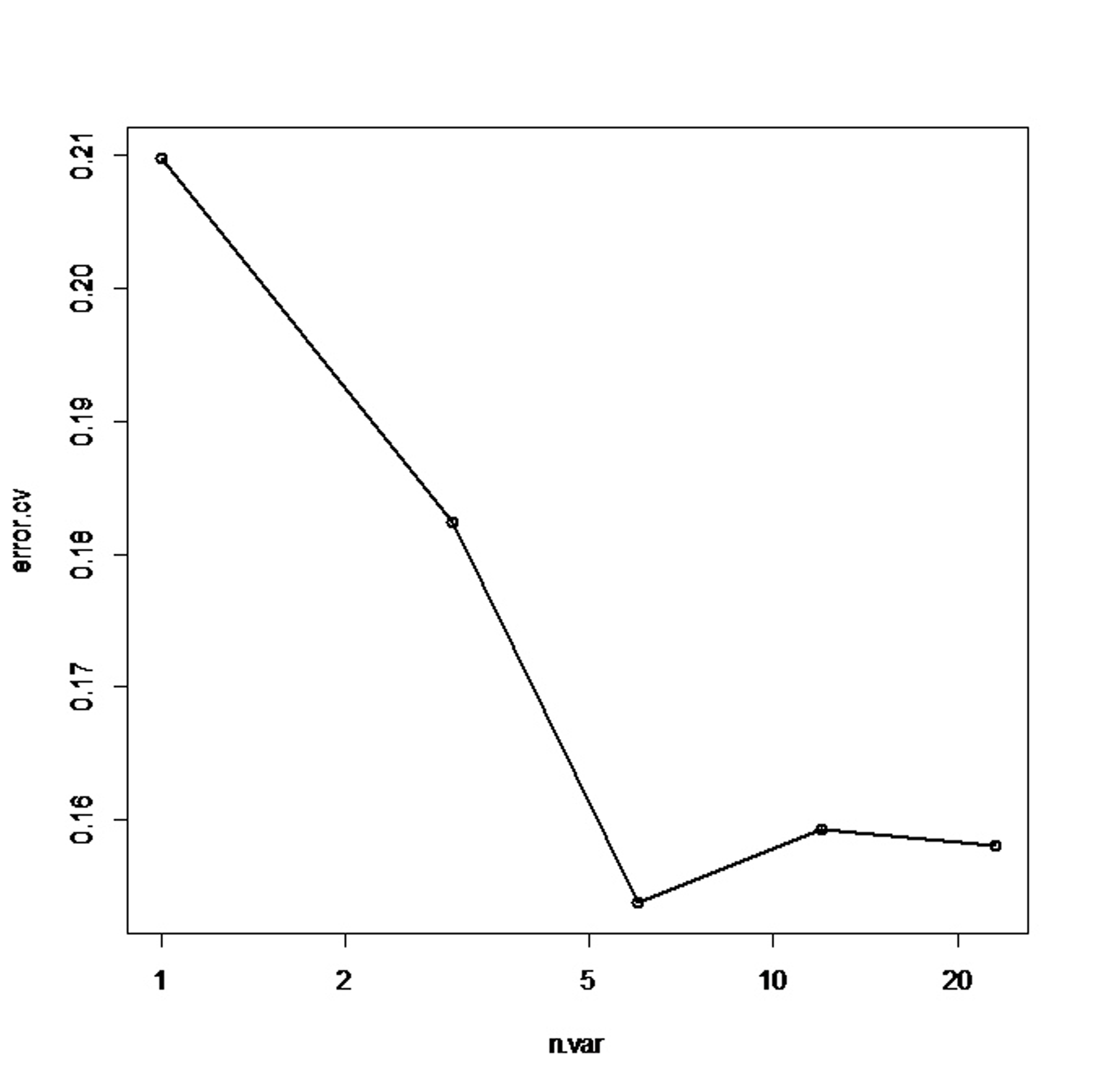

Supplement: S1 Fig — (TIF) [file pone.0164570.s001.tif]

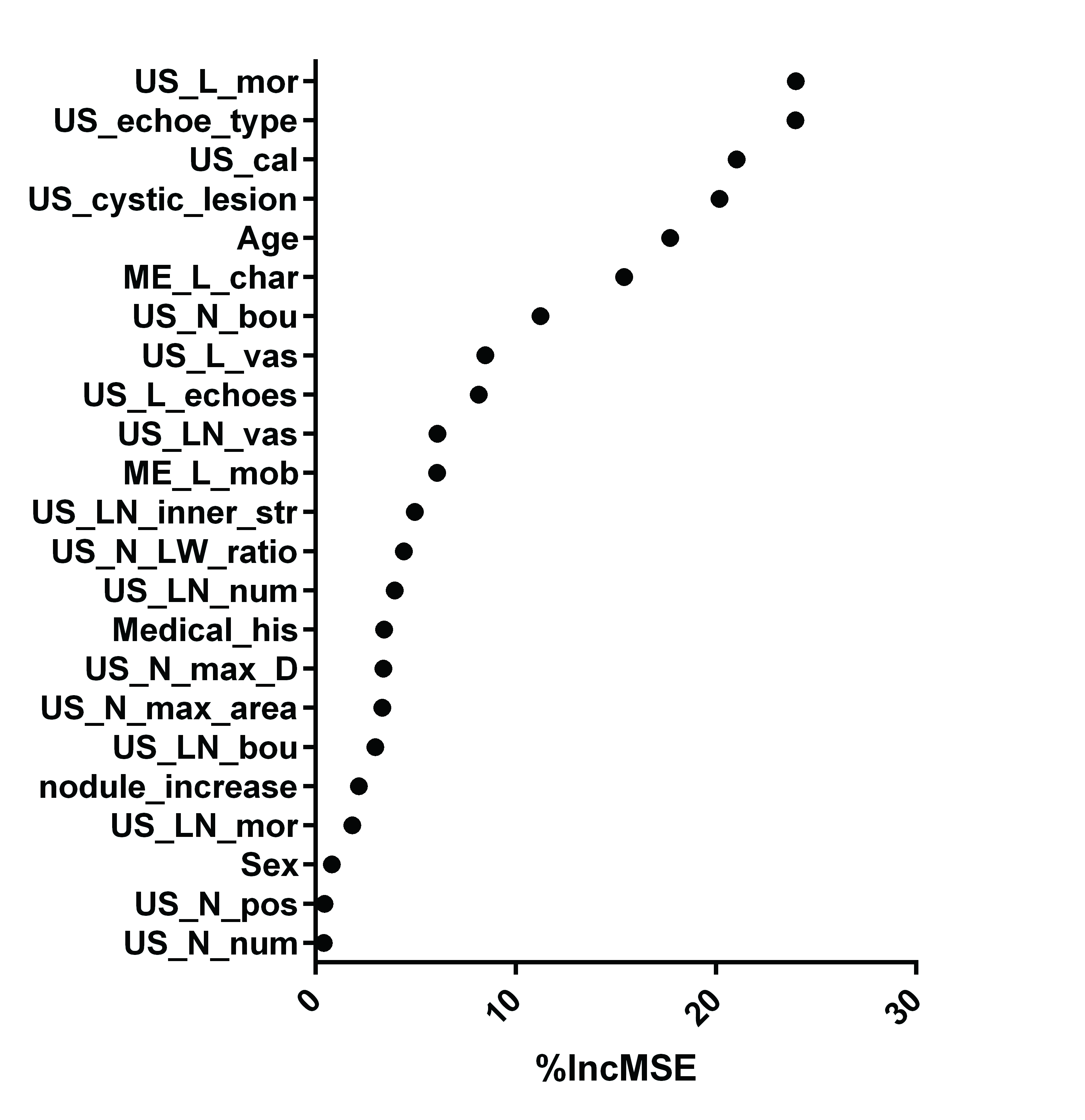

Supplement: S2 Fig — (TIF) [file pone.0164570.s002.tif]
