# Supplementary material for: Classification of Benign and Malignant Thyroid Nodules Using a Combined Clinical Information and Gene Expression Signatures
Source: PLoS One. 2016 Oct 24;11(10):e0164570. doi: 10.1371/journal.pone.0164570 (PMC5077123; doi:10.1371/journal.pone.0164570)
Supplement: S1 File — Table A. Histopathological subtype distribution in three cohorts. Cohort 1 comprised 711 patients from Renji Hospital with clinical information. Cohort 2 comprised 70 patients from Renji Hospital with clinical and gene expression information. Cohort 3 comprised 72 patients from Xinhua Hospital with clinical and gene expression information. Table B. Summary of the qPCR results for the expression of DPP4, SCG5 and CA12 in Cohort 2 and Cohort 3. (DOCX) [file pone.0164570.s003.docx]

**Table A. Histopathological subtype distribution in three cohorts.**

|  | **Cohort 1*** | **Cohort 2∫** | **Cohort 3∮** |
| --- | --- | --- | --- |
|  | No. of Nodules (%) | | |
| **Malignant** |  |  |  |
| Papillary thyroid carcinoma | 410(53.2) | 29(41.5) | 32(44.4) |
| Follicular thyroid carcinoma | 9(1.2) | 1(1.4) | 1(1.4) |
| Squamous cell thyroid carcinoma | 1(0.1) | 0(0.0) | 0(0.0) |
| Medullary thyroid cancer | 4(0.5) | 0(0.0) | 0(0.0) |
| Anaplastic thyroid carcinoma | 2(0.2) | 1(1.4) | 1(1.4) |
| **Benign** |  |  |  |
| Follicular adenomas | 227(29.5) | 20(28.6) | 23(32.0) |
| Thyroid hyperplasias | 52(6.8) | 8(11.4) | 8(11.1) |
| Hashimoto thyroiditis | 28(3.6) | 11(15.7) | 5(6.9) |
| Colloid nodule | 27(3.5) | 0(0.0) | 1(1.4) |
| Subacute thyroiditis | 7(0.9) | 0(0.0) | 1(1.4) |
| Hurthle cell adenoma | 4(0.5) | 0(0.0) | 0(0.0) |

*Cohort 1 comprised 711 patients from Renji Hospital with clinical information.

**∫**Cohort 2 comprised 70 patients from Renji Hospital with clinical and gene expression information.

**∮**Cohort 3 comprised 72 patients from Xinhua Hospital with clinical and gene expression information.

**Table B. Summary of the qPCR results for the expression of *DPP4*, *SCG5* and *CA12* in two cohorts.**

|  | Cohort 2 | | | Cohort 3 | | |
| --- | --- | --- | --- | --- | --- | --- |
|  | Mean of B ^a^ | Mean of M | *P*  Value ^b^ | Mean of B | Mean of M | *P*  Value ^c^ |
| DPP4 | 0.205$\pm$0.232 | 2.374$\pm$4.423 | 0.003 | 1.439$\pm$1.186 | 4.485$\pm$4.419 | <0.000 |
| SCG5 | 1.469$\pm$1.397 | 43.346$\pm$82.842 | 0.002 | 1.017$\pm$0.589 | 3.712$\pm$2.775 | <0.000 |
| CA12 | 0.568$\pm$0.667 | 0.733$\pm$1.193 | NS | 0.364$\pm$0.614 | 0.209$\pm$0.219 | NS |

B, benign thyroid nodules; M, malignant thyroid nodules; NS, not statistically significant (P>0.05).

a. The value of 2^-ΔCt^×100 was employed to measure the relative gene expression.

b. *P* values were determined by two-tailed Student’s t test for independent samples with 68 degrees of freedom.

c. *P* values were determined by two-tailed Student’s t test for independent samples with 70 degrees of freedom.
